# Spatial Distribution and Compartmental Allocation of Microplastics in Belowground Systems of Mulched *Phyllostachys violascens* Forests Along Urban–Rural Gradients

**DOI:** 10.3390/plants15111690

**Published:** 2026-05-30

**Authors:** Gang Lu, Zhukan Chen, Lili Fan, Liangjin Yao, Xiaoxia Zhou, Jingxiang Xu, Jiamei Chen, Jie Yang

**Affiliations:** 1 Zhejiang Forestry Fund Management Center, Hangzhou 310012, China; 13738037801@163.com; 2 Forestry and Agricultural Resources Protection Center of Fuyang District, Hangzhou 311400, China; 826026380@163.com; 3 Research Institute of Subtropical Forestry, Chinese Academy of Forestry, Hangzhou 311400, China; yfllyin@163.com; 4 Zhejiang Academy of Forestry, Hangzhou 310023, China; lj890caf@163.com; 5 Dongyang Forest Resources Comprehensive Service Center, Jinhua 321000, China; 1433141550@163.com; 6 Dongyang Forest Farm, Jinhua 321000, China; 48526797@163.com; 7 Fujian Academy of Forestry, Fuzhou 350012, China; maychen0805@126.com

**Keywords:** mulched bamboo forest, urban–rural gradient, microplastic pollution, rhizome–root system, migration pattern, ecological risk

## Abstract

Intensive management practices promote microplastic (MP) accumulation in *Phyllostachys violascens* forests, posing potential threats to ecosystem stability and the edibility safety of bamboo shoots. However, how urbanization and mulching duration jointly regulate MP distribution, transfer, and associated food safety risks remains unclear. We investigated MP dynamics across urban–rural gradients (suburban vs. exurban) under different mulching durations (no mulching, short–term mulching, and long–term mulching), focusing on the rhizome–root–soil system and bamboo shoots. MP abundance was significantly higher in suburban forests, with maxima under long–term mulching, whereas in exurban forests, peaks occurred under long–term mulching. Urbanization also altered MP allocation patterns, with enrichment in rhizome roots and rhizomes in suburban forests but greater accumulation in bamboo shoots in exurban forests. Long–term mulching markedly enhanced MP accumulation across all components, particularly in clump roots, where abundance was three times higher than that in exurban forests. MPs were predominantly small (20–50 μm), mainly composed of acrylates (ACRs), polyvinyl chloride (PVC), polypropylene (PP), polyethylene terephthalate (PET), polyurethane (PU), and polyethylene (PE). Along the “soil–root–rhizome” continuum, contrasting transfer patterns emerged between suburban and exurban forests, with soil total potassium identified as the key driver regulating MP migration and redistribution. Although the pollution load index indicated moderate contamination without significant accumulation in bamboo shoots, the ecological risk index revealed a high ecological risk, highlighting potential food safety concerns. Overall, MP accumulation and migration in *Ph. violascens* systems are jointly shaped by urban–rural gradients and mulching duration, with implications for belowground processes and the safety of edible bamboo shoots.

## 1. Introduction

In recent years, microplastics (MPs; <5 mm), as emerging environmental contaminants, have attracted increasing attention regarding their environmental persistence and potential ecological risks [1]. Within soil–plant systems, MPs can influence multiple biological processes, including soil microbial activity, root development, and plant physiological performance [2,3]. Previous studies have demonstrated that MPs can inhibit microbial metabolic activity, disrupt root growth, and consequently impair normal plant growth and reproduction [3]. In addition, nanoscale MPs (<100 nm) can be transported from soil into the vascular system via transpiration–driven flow, facilitating their accumulation in edible organs and raising concerns about food safety and human health risks [2].

*Phyllostachys violascens*, an economically important bamboo species cultivated for edible shoots in China, relies heavily on mulching management to enhance productivity and economic returns [4,5]. However, long–term mulching practices may introduce substantial quantities of exogenous MPs into bamboo forests. Moreover, major cultivation regions are typically located in densely populated and highly urbanized areas, characterized by intensive transportation networks and sand transportation networks. In such contexts, MPs can enter bamboo forest ecosystems via multiple pathways, including wastewater discharge, sewage irrigation, and atmospheric deposition [6]. These inputs contribute to elevating MP accumulation in soils and increase the likelihood of MP migration from soil to bamboo systems, particularly within the rhizome–root–soil continuum and edible bamboo shoot tissues [7].

MP uptake and internal transport in plants have been increasingly documented. MPs can enter plant tissues through root epidermis uptake or through discontinuities formed during root development [8,9]. Once internalized, particles can be translocated via the vascular system, primarily driven by transpiration flow. Although several uptake pathways (e.g., endocytosis, membrane transport, and entry through root structural gaps) have been proposed, their relative importance under field conditions remains uncertain and likely depends on particle size and plant physiological traits [10]. Overall, the current understanding of MP transfer within plant systems remains limited, particularly in complex agroforestry ecosystems where multiple environmental and biological factors interact.

In this study, we defined the urban–rural gradient as a continuum from suburban (high anthropogenic disturbance, including intensive management and pollution inputs) to exurban (relatively low disturbance and background conditions). Using *Ph. violascens* forests with different mulching durations, we investigated MP distribution and transfer within the rhizome–root–soil system and bamboo shoots. Specifically, we aimed to (i) characterize MP distribution patterns across belowground components and edible tissues, (ii) quantify MP transfer along the soil–plant continuum, and (iii) identify the key roles of root traits, soil properties, and MP characteristics in regulating these processes. The findings aim to provide new insights into the interaction between urbanization, management practices, and MP dynamics, with implications for ecological risk assessment and food safety in agroforestry systems.

## 2. Results

### 2.1. Abundance and Bioaccumulation of MPs in Rhizome–Root Systems and Bamboo Shoots of Experimental Ph. violascens Forests

**Figure 1:**
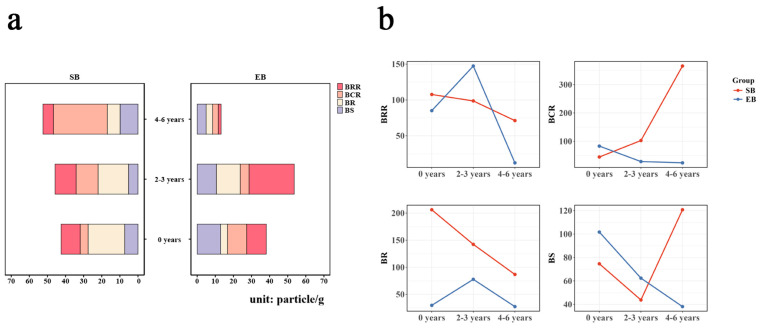
Abundance of MPs and the bioaccumulation factor (BCF) in the rhizome–root system and bamboo shoot components of *Phyllostachys violascens*. (**a**) MP abundance; (**b**) Bioaccumulation factor. Note: SB, suburban bamboo forest; EB, exurban bamboo forest; 0 years, no mulching; 2–3 years, short–term mulching; 4–6 years, long–term mulching; BRR, bamboo rhizome root; BCR, bamboo clump root; BR, bamboo rhizome; and BS, bamboo shoot. The same abbreviations apply throughout the manuscript.

As shown in Figure 1a, the MP abundance values in rhizome–root systems and bamboo shoots were consistently higher in suburban forests than in exurban forests across all mulching durations. Under no and short–term mulching, the differences in MP accumulation between forest types were relatively small. However, under long–term mulching, the disparity became pronounced, with MP abundance in suburban forests reaching nearly threefold higher than in exurban forests. With increasing mulching duration, MP abundance in suburban forests showed a continuous increase, reaching a maximum under long–term mulching. In contrast, exurban forests showed an initial increase followed by a decline, with the lowest values under short–term mulching.

Significant differences in MP distribution were observed among bamboo components. In suburban forests, MPs were primarily accumulated in rhizomes and rhizome roots under no–mulching conditions; in rhizome roots, clump roots, and rhizomes under short–term mulching; and mainly in clump roots and bamboo shoots under long–term mulching. In exurban forests, MPs accumulated in rhizome roots, clump roots, and bamboo shoots under no–mulching conditions; in rhizome roots, rhizomes, and bamboo shoots under short–term mulching; and showed a marked overall decline across all components under long–term mulching, accompanied by reduced inter–component differences.

The bioaccumulation factor (BCF) results indicated that the effects of urban–suburban distance on MP accumulation varied with mulching duration (Figure 1b). In suburban forests, the bioaccumulation capacity of rhizomes and rhizome roots for MPs decreased continuously with increasing mulching duration. In exurban forests, it increased initially and then decreased. For bamboo shoots and clump roots, the BCF declined steadily in exurban forests, whereas in suburban forests, it increased continuously in bamboo shoots and showed a decrease followed by an increase in clump roots, resulting in an overall upward trend.

Overall, MP abundance was higher in suburban forests than in exurban forests. Mulching duration significantly influenced the distribution patterns of MPs across rhizome root systems and bamboo shoot components. Under long–term mulching, MP bioaccumulation capacity in exurban forests declined across all components, whereas in suburban forests, it decreased in rhizomes and rhizome roots but increased in bamboo shoots and clump roots.

### 2.2. Characteristics of MP Accumulation in Rhizome–Root Systems and Bamboo Shoots of Experimental Ph. violascens Forests

**Figure 2:**
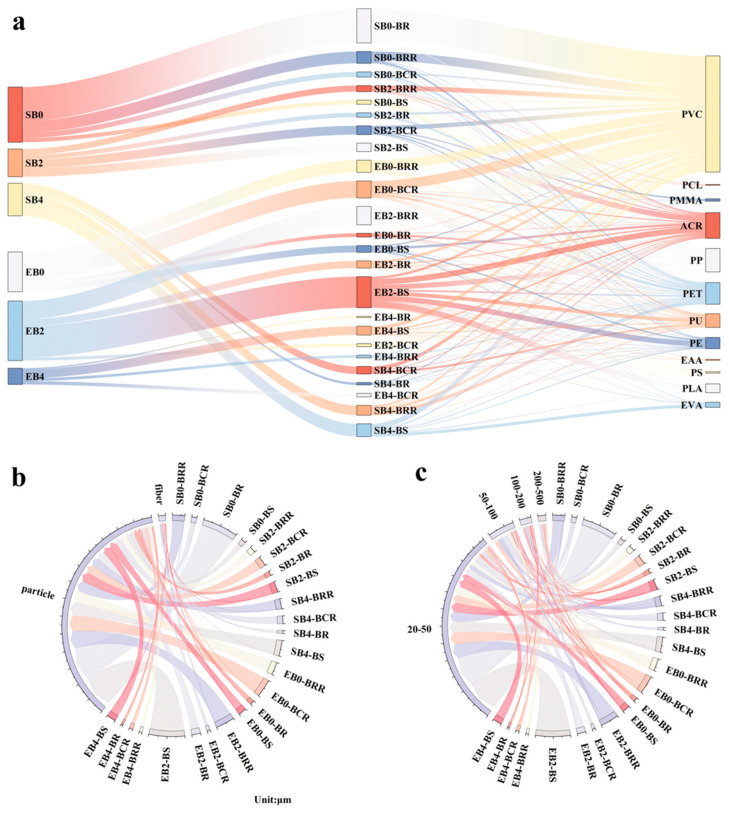
Characteristics of MPs in the rhizome–root system and bamboo shoot components of *Ph. violascens*. (**a**) MP polymer types (PP, polypropylene; EAA, ethylene–acrylic acid copolymer; ACR, polyacrylate; PET, polyethylene terephthalate; PS, polystyrene; PU, polyurethane; PE, polyethylene; PMMA, polymethyl methacrylate; PLA, polylactic acid; PVC, polyvinyl chloride; EVA, ethylene–vinyl acetate copolymer; and PCL, polycaprolactone); (**b**) MP shapes (particle: granular form; fiber: fibrous form); and (**c**) MP size classes.

A total of 12 MP polymer types were in rhizome–root systems and bamboo shoot components across both forest types, with clear but simplified compositional patterns (Figure 2a). Among them, polyethylene terephthalate (PET), polyethylene (PE), polypropylene (PP), polyvinyl chloride (PVC), and polyacrylate (ACR) were consistently dominant, showing higher detection frequencies and abundances than other polymers. Although minor polymers exhibited sporadic and component–specific occurrences, their overall contributions were limited. Notably, exurban forests showed a relatively higher diversity and abundance of MPs, particularly for certain polymers such as PE in bamboo shoots. Despite variations in mulching duration, the dominant polymers displayed highly consistent distribution patterns across components, suggesting stable transport and allocation processes within the system.

In terms of morphology (Figure 2b), MPs were overwhelmingly dominated by particulate forms (97%), while fibrous MPs occurred infrequently and were mainly restricted to belowground components, indicating a highly heterogeneous distribution pattern. Regarding particle size (Figure 2c), the 20–50 μm range was widely distributed across all components. Larger particles (>200 μm) were rare, particularly in bamboo shoots, and their occurrence decreased with increasing mulching duration. This pattern indicates that mulching, especially long–term mulching, limited the migration of larger MPs into plant–associated compartments.

Overall, MPs in rhizome–root systems and bamboo shoots were characterized by dominance of a few major polymer types, particulate morphology, and relatively small particle sizes. While urban–suburban distance and mulching duration influenced MP abundance, their effects on overall compositional patterns were limited, reflecting the consistent source and transport pathway of MPs within the system.

### 2.3. Root Morphological Characteristics of Experimental Ph. violascens Forests

Both forest type and mulching duration significantly influenced root morphological development (Table 1). In suburban forests, clump roots and rhizome roots exhibited distinctly different adaptive strategies. Clump roots showed a pronounced “thin–and–elongated” trend with increasing mulching duration: total root length and specific root length increased significantly (by more than 50% under long–term mulching compared with no mulching), whereas average root diameter, root volume, and root surface area decreased markedly (by approximately 50%). Root tip number and branching number also declined. In contrast, rhizome roots exhibited a more complex response to mulching duration, characterized by structural reconfiguration. Total root length, root tip number, and branching number reached their maximum values under long–term mulching, indicating enhanced elongation and branching capacity. However, average diameter and root volume peaked under short–term mulching.

In exurban forests, root responses to mulching duration were more pronounced, and a competitive relationship between clump roots and rhizome roots was evident. All morphological traits of clump roots increased significantly under long–term mulching; total root length, root tip number, branching number, and crossing number all increased by more than 200% compared with the no–mulching treatment. In contrast, rhizome roots exhibited optimal development under short–term mulching, with total root length, surface area, root tip number, and branching number reaching their maximum values, followed by a significant decline under long–term mulching.

Overall, the root growth of *Ph. violascens* responded strongly to both forest type and mulching duration. In suburban forests, root development was characterized by a relatively coarse and robust growth form under no mulching, whereas long–term mulching promoted a shift toward a more “thin–and–elongated” strategy in clump roots. In exurban forests, root systems generally exhibited enhanced elongation under mulching, with particularly strong overall development of clump roots under long–term mulching.

### 2.4. Translocation Factors (TFs) and Driving Mechanisms of MPs in Rhizome–Root Systems and Bamboo Shoots of Experimental Ph. violascens Forests

**Figure 3:**
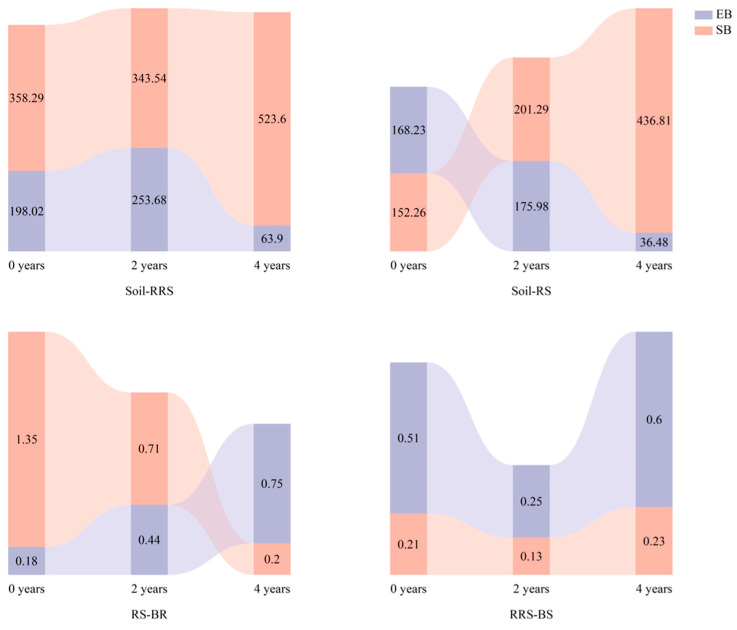
Translocation factors (TFs) of MPs in the rhizome–root system and bamboo shoot components of *Ph. violascens*. Note: SB, suburban bamboo forest; EB, exurban bamboo forest; 0 years, no mulching; 2–3 years, short–term mulching; 4–6 years, long–term mulching; Soil–RRS, soil to rhizome–root system; Soil–RS, soil to root system; RS–BR, root system to bamboo rhizome; RRS–BS, rhizome–root system to bamboo shoot.

As shown in Figure 3, the translocation capacity of MPs along different transport pathways was significantly regulated by both forest type and mulching duration. In the “soil–rhizome–root system” pathway results, suburban forests exhibited a stronger translocation capacity than exurban forests. With increasing mulching duration, MP translocation in suburban forests first decreased and then increased, whereas in exurban forests it showed an initial increase followed by a decline. Notably, the magnitude of variation (i.e., the difference between the minimum and maximum values) increased markedly from short–term to long–term mulching in both forest types.

In the “soil–total root system” pathway results, opposite temporal trends were observed between the two forest types. In suburban forests, the translocation capacity continuously increased with mulching duration and reached its maximum under long–term mulching. In contrast, exurban forests showed an initial increase followed by a decline, with the lowest value observed under long–term mulching.

In the “total root system–bamboo rhizome” pathway results, the two forest types again exhibited contrasting trends, but in a reversed pattern. The translocation capacity in suburban forests decreased progressively with increasing mulching duration, whereas it increased continuously in exurban forests.

In the “rhizome–root system–bamboo shoot” pathway results, both forest types exhibited a similar non–linear pattern characterized by an initial decrease followed by an increase. However, the fluctuation was more pronounced in exurban forests, whereas suburban forests showed relatively moderate variation.

**Figure 4:**
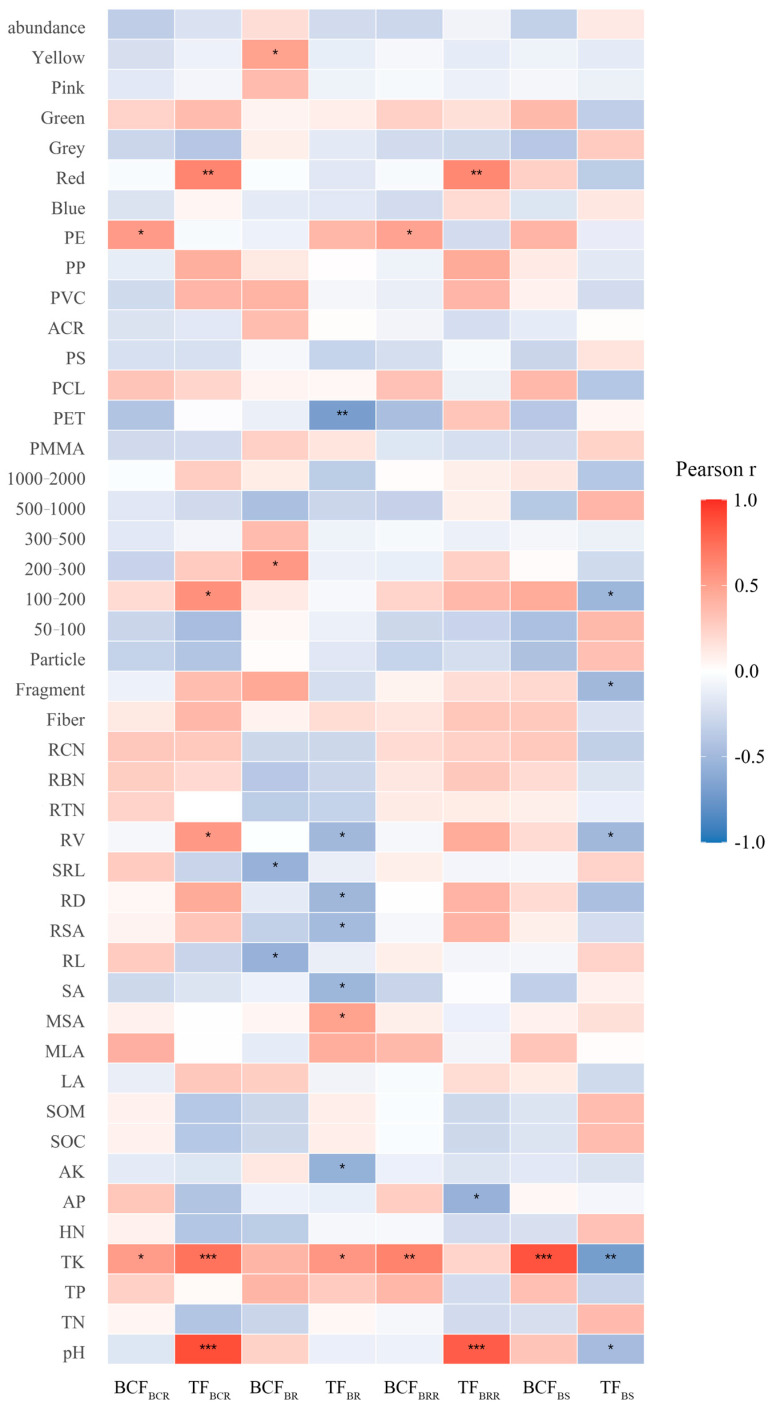
Heatmap of Pearson correlation analysis between the values of the BCF and TFs and environmental and biological factors in the rhizome–root system and bamboo shoot components of *Ph. violascens*. Note: (1) Pearson correlation coefficients were calculated based on standardized variables. (2) Statistical significance was assessed using two–tailed correlation tests, where * indicates *p* < 0.05, ** indicates *p* < 0.01, and *** indicates *p* < 0.001. Abundance, soil MP abundance; soil MP color (Yellow, Pink, Green, Grey, Red, and Blue); soil MP polymer types (ABS, PE, PP, PVC, ACR, PS, PCL, PET, and PMMA); soil MP particle size (1000–2000, 500–1000, 300–500, 200–300, 100–200, and 50–100 μm); soil MP morphology (Particle, Fragment, and Fiber); root morphological traits (RCN, root crossing number; RBN, root branching number; RTN, root tip number; RV, root volume; SRL, specific root length; RD, root diameter; RSA, root surface area; and RL, root length); soil physical properties (SA, small aggregate; MSA, medium–small aggregate; MLA, medium–large aggregate; and LA, large aggregate); soil chemical properties (SOM, soil organic matter; SOC, soil organic carbon; AK, available potassium; AP, available phosphorus; HN, hydrolyzable nitrogen; TK, total potassium; TP, total phosphorus; TN, total nitrogen; and pH, soil pH).

To further elucidate the effects of various factors on MP accumulation and translocation in different bamboo components, Pearson correlation analyses were performed between soil chemical properties, soil physical properties, root morphological traits, soil MP characteristics, and the values of the BCF and TFs in different bamboo components. The results are presented in Figure 4.

The correlation analysis indicated that only soil total potassium (TK) and PE–type MPs were significantly and positively correlated with the BCF in clump roots (*p* < 0.05). Overall, soil chemical and physical properties, as well as root morphological traits, generally exerted positive effects on the BCF, whereas MP characteristics in soil—including morphology, particle size, color, polymer type, and abundance—showed overall negative effects. In contrast, the TF exhibited stronger and more variable responses to environmental and biological variables. Soil pH and TK were highly significantly and positively correlated with the TF (*p* < 0.001), while red MPs showed a significant positive correlation (*p* < 0.01). Root volume and MPs in the 100–200 μm size class also showed significant positive correlations (*p* < 0.05). Compared with the BCF, soil chemical and physical properties generally exerted negative effects on the TF, whereas root morphological traits showed positive effects. The influences of MP characteristics were inconsistent among indicators, while overall MP abundance showed a negative relationship with the TF.

For rhizome–root systems, similar patterns were observed. Only soil TK and PE–type MPs showed significant positive correlations with the BCF, with TK reaching a highly significant level (*p* < 0.01). The effects of soil chemical and physical properties were variable, whereas root morphological traits and MP morphology generally showed positive effects. In contrast, MP particle size, color, polymer type, and abundance generally exhibited negative effects on bioaccumulation. For the TF, multiple root morphological traits were significantly negatively correlated (*p* < 0.05), while soil TK and medium–small aggregate showed significant positive correlations (*p* < 0.05), and PET–type MPs showed a significant negative correlation (*p* < 0.01). Overall, most variables other than soil chemical properties were negatively correlated with the TF.

For bamboo rhizomes, the BCF was significantly negatively correlated with specific root length and total root length (*p* < 0.05). The effects of soil chemical properties were inconsistent, while soil physical properties tended to exert negative effects. Root morphological traits and most MP characteristics (morphology, particle size, color, and polymer type) generally showed negative effects, whereas MP abundance showed a positive effect. Regarding the TF, multiple root traits were significantly negatively correlated (*p* < 0.05), while soil TK and medium–small aggregate showed significant positive correlations (*p* < 0.05), and PET–type MPs showed a significant negative correlation (*p* < 0.01). Overall, most variables except soil chemical properties were negatively associated with the TF.

For bamboo shoots, the BCF showed a strong positive correlation with soil TK (*p* < 0.001), with TK reaching a significant level (*p* < 0.01). The effects of soil chemical properties, soil physical properties, and MP characteristics were variable, while root morphological traits generally showed positive effects, and MP abundance showed an overall negative effect. For the TF, soil TK was significantly negatively correlated (*p* < 0.01), while soil pH, root volume, fragmented MPs, and 100–200 μm MPs were significantly negatively correlated (*p* < 0.05). Overall, soil chemical and physical properties and MP characteristics showed inconsistent effects, root morphological traits exerted negative influences, and MP abundance showed a positive relationship.

### 2.5. Safety Assessment of MPs in Bamboo Shoots from Mulched Ph. violascens Forests

As shown in Table 2, the ecological risk index (H) of MPs in bamboo shoots across different experimental forest types was generally at a relatively high level, ranging from 639.63 to 7501.25. Except for the short–term mulching treatment in suburban forests, which exhibited a Class III ecological risk, all other treatments reached Class IV ecological risk, indicating that MPs in bamboo shoots pose a high potential ecological risk in terms of polymer toxicity.

In suburban forests, the H values of MPs in bamboo shoots under no mulching and long–term mulching were 2861.86 and 5718.68, respectively, both significantly higher than that under short–term mulching (639.63), suggesting that prolonged mulching may increase the proportion of high–risk polymers in bamboo shoots. A similar trend was also observed in exurban forests, where the highest H value occurred under long–term mulching (6876.81), while short–term mulching showed relatively lower values (1561.27).

The differences in pollution load index (PLI) among different mulching durations within both suburban and exurban forests were relatively small (0.2–0.4), and all treatments fell within the moderate pollution level, with no cases of light or severe pollution observed. However, increasing mulching duration did not lead to a consistent upward trend in PLI values. In suburban forests, the PLI of bamboo shoots under long–term mulching (1.41) was higher than that under short–term mulching (1.02), whereas in exurban forests, the PLI under long–term mulching was lower than that under short–term mulching.

Overall, MP contamination in bamboo shoots from mulched *Ph. violascens* forests exhibited a combined pattern of “high H index–moderate PLI”. The ecological risk was predominantly at Class IV level, while the overall pollution level remained moderate.

## 3. Discussion

### 3.1. Effects of Urban–Suburban Distance on MP Abundance and Distribution in Rhizome–Root Systems and Bamboo Shoots

Urban–suburban distance is a key spatial factor regulating MP abundance and distribution patterns in rhizome–root systems and bamboo shoot components of mulched *Ph. violascens* forests. Overall MP abundance in suburban forests was significantly higher than that in exurban forests, with differences reaching up to threefold under long–term mulching. This indicates that proximity to urban areas exerts a strong influence on MP accumulation. Suburban forests are typically exposed to more complex and continuous external MP inputs, including residues from agricultural mulching materials, traffic–derived wear particles, and plastic debris from domestic sources [11,12]. Such persistent inputs contribute to elevated environmental background levels, thereby promoting the continuous accumulation of MPs within bamboo forest ecosystems [13].

At the polymer level, although multiple MP types were detected in both suburban and exurban forests, spatial differences in their distribution were still evident. PCL was detected exclusively in suburban forests, whereas PS was found only in exurban forests, reflecting differences in regional source inputs. In contrast, several polymer types, including PVC, PP, PET, and ACR, were consistently detected in both forest types and exhibited similar distribution patterns along the rhizome–root–bamboo shoot transport pathway. This suggests that these polymers may follow relatively stable internal transport routes within bamboo plants and are more readily absorbed and translocated than other MP types.

In terms of morphology and particle size, urban–suburban distance exerted a relatively limited influence on MP distribution patterns. Particulate MPs dominated in both forest types, whereas fibrous MPs were mainly restricted to rhizome roots and clump roots, indicating the limited mobility of fibrous particles within plant tissues. This finding is consistent with previous studies suggesting that MPs with smaller size and more regular shapes are more easily absorbed through root surfaces and transported within plants [14]. In addition, MPs in the 20–50 μm size range were widely detected across all components in both forest types, whereas the detection frequency of particles larger than 200 μm decreased significantly with increasing mulching duration, further supporting the presence of strong size–selective uptake and translocation processes in plant tissues [15,16].

### 3.2. Effects of Mulching Duration on MP Abundance and Distribution in Rhizome–Root Systems and Bamboo Shoots

Mulching duration significantly regulated MP accumulation levels and distribution patterns in rhizome–root systems and bamboo shoot components. In suburban forests, MPs exhibited clear component–specific migration patterns across different mulching stages. Under no–mulching conditions, MPs were mainly accumulated in bamboo rhizomes and rhizome roots. With short–term mulching, accumulation expanded to clump roots, and under long–term mulching, MPs were further transferred to clump roots and bamboo shoots. These results indicate that, in suburban forests, increasing mulching duration not only promotes continuous MP accumulation in belowground components but may also facilitate upward migration along the soil–total root system–bamboo shoot pathway.

Previous studies have shown that under high environmental contamination loads, healthy plant root systems can facilitate MP entry into vascular tissues through root fissures or endodermal pathways driven by transpiration, enabling subsequent translocation to aboveground organs [16,17]. In addition, mulching material transport, installation, and turnover processes have been reported to increase the risk of plastic incorporation into soils while accelerating mechanical fragmentation and aging of plastics, thereby indirectly elevating the environmental background levels of MPs in soils [18]. Therefore, under long–term mulching, even if MP uptake rates by bamboo culms remain relatively stable, MP abundance in rhizome–root systems and bamboo shoots may still increase synchronously with mulching duration.

In contrast, exurban forests showed an opposite pattern, with MP accumulation in bamboo components generally decreasing under long–term mulching. Moreover, differences in MP abundance among components were substantially reduced, indicating a more homogeneous distribution and a weakened internal transport and allocation process. This phenomenon may be associated with soil acidification, nutrient imbalance, and a decline in overall soil ecological health in exurban forests [19]. Under long–term mulching, rhizome–root systems may experience prolonged physiological stress or decline, resulting in reduced capacity for MP uptake and translocation. Even when MPs enter the rhizome–root system, they may be preferentially redistributed toward bamboo culms or leaves rather than accumulating locally. The combined effects of these processes likely explain the sharp decline in MP abundance across bamboo components in exurban forests under long–term mulching conditions.

### 3.3. Safety Assessment of Bamboo Shoots in Mulched Ph. violascens Forests

Across different urban–suburban gradients, the H index and PLI index of MPs in bamboo shoots exhibited clearly asynchronous change patterns, indicating that the potential dietary safety risk of MPs in bamboo shoots is determined not only by MP abundance but also, more importantly, by polymer composition and structural characteristics.

The relatively high H index primarily reflects the dominance of high–hazard–score polymer types in bamboo shoots. Previous studies have shown that different polymers vary substantially in the types of additives used during production and their inherent toxicological properties. Even at low abundance, the accumulation of high–risk polymers can significantly elevate ecological risk levels [20]. This finding is consistent with the “moderate pollution but high ecological risk” pattern observed in this study, suggesting that high–risk polymers may exert a certain influence on the edible safety of bamboo shoots. In both suburban and exurban forests, the H index under long–term mulching was higher than that under short–term mulching, indicating that prolonged mulching may increase the probability of high–risk polymer accumulation in bamboo shoots. However, the PLI index did not increase synchronously with mulching duration, suggesting that long–term mulching does not necessarily promote an increase in the total MP abundance in bamboo shoots. This further confirms that the elevated H index is driven by changes in polymer composition rather than by increases in MP abundance.

In addition, the H and PLI indices assess MP risk from two complementary perspectives, polymer toxicity and abundance, respectively, providing a more comprehensive and reliable framework for evaluating MP–related risks in bamboo shoots. Although the overall MP contamination level in bamboo shoots remains moderate and is unlikely to pose an immediate threat to food safety, the associated ecological risks should not be overlooked. Particular attention should be given to long–term mulching systems, where such risks may become more pronounced.

## 4. Materials and Methods

### 4.1. Study Area

The study was conducted in Lin’an District, Hangzhou City (30°23′ N, 119°72′ E), China. This region has a typical subtropical monsoon climate, with a mean annual temperature of 15–18 °C and an annual precipitation of 1628 mm. Precipitation and temperature are seasonally synchronized from April to September, characterized by hot and humid conditions that are highly suitable for the growth and development of *Ph. violascens*.

Lin’an District is one of the principal production areas of *Ph. violascens* in China. According to statistics from 2022, the cultivation area reached 2.86 × 10^5^ mu, with an annual fresh shoot yield of 4.5 × 10^5^ tons, ranking first nationwide in terms of industrial scale. A comprehensive industrial chain has been established locally, encompassing seedling propagation, large–scale cultivation, intensive processing, and ecological tourism. Core production areas, such as Taihuyuan Town, supply more than 60% of the bamboo shoot demand in the Yangtze River Delta region [6].

### 4.2. Experimental Design

Mulched *Ph. violascens* forests were selected in both suburban (SB, 6 km from the urban center) and exurban (EB, 16 km from the urban center) areas within the study region. In each forest type, three mulching–duration treatments were established: no mulching (0 years), short–term mulching (2–3 years), and long–term mulching (4–6 years). For each treatment, six replicate plots (10 m × 10 m) were established, with a minimum of 10 m between plots to ensure spatial independence.

### 4.3. Plant Sample Collection

#### 4.3.1. Rhizome–Root Sampling

In each plot of the experimental bamboo forests, five healthy *Ph. violascens* individuals aged 2–3 years were selected, resulting in a total of 30 plants per site type. Each selected plant was carefully excavated from the base, and the rhizome, clump root, and rhizome root components were separated. Samples from the same site type were composited for subsequent analysis. To prevent exogenous contamination, particularly from MPs, all samples were handled using hemp ropes or kraft paper bags, and contact with plastic materials was strictly avoided throughout the sampling and transport processes.

#### 4.3.2. Bamboo Shoot Sampling

During the shooting period of *Ph. violascens*, bamboo shoots were collected from each plot by random excavation. At least 60 shoots were collected per site type (≥10 shoots per plot) and transported to the laboratory in paper boxes. From each site type, 40 shoots with similar size and intact morphology were selected and pooled to form composite samples for further analysis.

### 4.4. Measurement of Root Morphological Traits

Rhizome–root and clump root samples were thoroughly washed, and any adhering plastic debris was carefully removed from the surface. Cleaned samples were then spread evenly and scanned using a flatbed scanner. After scanning, the root samples were placed in paper envelopes for preservation. The scanned images were analyzed using the WinRHIZO 2016a root analysis system (Regent Instruments, Quebec, QC, Canada) to obtain key root morphological parameters, including total root length (TL), root surface area (RSA), root diameter (RD), specific root length (SRL), root volume (RV), root tip number (RTN), root branching number (RBN), and root crossing number (RCN).

### 4.5. Extraction and Identification of MPs in Plant Samples

Rhizome root, clump root, and rhizome samples were thoroughly washed, and any plastic debris adhering to the surface was carefully removed. For bamboo shoots, the outer sheaths were discarded, and only the edible tissues were retained, washed with deionized water, and homogenized by chopping. All samples were oven–dried at <60 °C, ground into powder using a grinding device, and sieved (4 mesh). A 5 g subsample was weighed into a 10 mL stoppered test tube, and sodium hydroxide solution (three times the sample volume) was added. The mixture was thoroughly stirred for approximately 10 min and then digested in a 60 °C water bath for 8–24 h to remove organic matter. The resulting supernatant was subjected to vacuum filtration using a 20 μm stainless steel membrane. The filter membrane was subsequently immersed in ethanol and ultrasonicated, and then removed and rinsed three times with ethanol. The ethanol rinsates were combined and concentrated in an oven at 60 °C to approximately 150 μL. The concentrated solution was then transferred dropwise onto a low–dispersion infrared reflection (LDIR) glass slide and allowed to dry completely before LDIR analysis [21].

To minimize contamination, plastic materials were strictly avoided throughout sample processing. All solutions were pre–filtered (13 μm polytetrafluoroethylene membrane) and stored in sealed containers. Glassware was thoroughly cleaned, combusted at high temperature, and rinsed three times with ethanol prior to use. Laboratory surfaces and the surrounding environment were maintained in a clean condition to reduce interference from airborne fibers.

MPs were identified, imaged, and quantified using a microscope (Olympus BX43, Olympus Corporation, Tokyo, Japan) to determine their abundance and morphological characteristics. MPs with particle sizes > 1 mm (<5 mm) were further analyzed for polymer composition using a Fourier–transform micro–infrared spectrometer (Nicolet iN10, Thermo Fisher Scientific, Waltham, MA, USA).

The identification criteria were as follows: (i) based on microscopic observations, MPs were classified into two morphological types: fibers (thin, elongated filaments) and particles (small, granular shapes); (ii) particle size classes, determined using a stereomicroscope, were categorized as 20–50 μm, 50–100 μm, 100–200 μm, and 200–500 μm; and (iii) polymer types were identified by matching spectra against a reference library (match > 60%). Polymers with ambiguous identification (e.g., rubber, resins, and polyamides) were excluded. The final polymer types identified included PP, EAA, ACR, PET, PS, PU, PE, PMMA, PLA, PVC, EVA, and PCL.

### 4.6. BCF and TF Values

The BCF value [22] was used to quantify the capacity of different bamboo components to accumulate MPs. Specifically, the BCF was calculated to evaluate MP accumulation in rhizome–root systems and bamboo shoot tissues:

(1) $$\mathrm{BCF}=\frac{M}{N}$$

where M represents the abundance of MPs in different bamboo components (rhizome–root, clump root, rhizome, and bamboo shoots), and N denotes the abundance of MPs in the soil.

The TF value [22] was used to assess the transport capacity of MPs among different bamboo components. The TF was calculated to evaluate the migration of MPs within the rhizome–root system and into bamboo shoots:

(2) $$\mathrm{TF}=\frac{X}{Y}$$

where X represents the abundance of MPs in a given bamboo component (rhizome–root, clump root, rhizome, or bamboo shoots), and Y denotes the abundance of MPs in the reference compartment, including soil, total root system (sum of clump root and rhizome–root), or bamboo shoots. These ratios respectively characterize MP transfer from soil to the rhizome–root system (rhizome root, clump root, and rhizome), from soil to the root system (rhizome root and clump root), from the total root system to the rhizome, and from the rhizome–root system to the bamboo shoots.

### 4.7. Safety Assessment of Bamboo Shoots

The potential risk of MPs in bamboo shoots was evaluated using two complementary indicators: polymer–based hazard characterization and MP abundance. The H and PLI indices were applied to assess the MP–related safety of bamboo shoots in the experimental *Ph. violascens* forests.

The H index was based on the polymer hazard scoring method proposed by Lithner et al. [23], which integrates the relative proportion of different polymers with their assigned hazard scores [24]. The calculation is shown in Equation (3):

(3) $$H=\Sigma P_{n}\times S_{n}$$

where H is the MP–related risk index; P_n_ represents the percentage of each MP polymer type at each sampling site; and S_n_ denotes the corresponding hazard score. The hazard scores of selected polymers are provided in the Supplementary Materials, Table S1. This index reflects the relative contribution of different polymer types to overall potential risk, rather than their direct biological toxicity.

The PLI index, based on MP abundance, was used to assess the relative pollution level of MPs. The calculation of this evaluation model is given in Equations (4)–(6):

(4) $$CF_{i}=\frac{C_{i}}{C_{\mathrm{oi}}}$$

(5) $${\mathrm{PLI}}_{n}=\sqrt{{\mathrm{CF}}_{i}}$$

(6) $$\mathrm{PLI}=\sqrt[{n}]{{\mathrm{PLI}}_{1}\times {\mathrm{PLI}}_{2}\times {\mathrm{PLI}}_{3}\times \cdots \times {\mathrm{PLI}}_{n}}$$

where CF_i_ is the contamination factor of MPs; C_0i_ is the background value of MP abundance. For soil samples, the lowest MP abundance detected among all soil samples in this study was used as the background value (items·kg^−1^) [21]; the soil MP data used in this study are presented in the Supplementary Materials, Figure S1, while for plant samples, the lowest MP abundance detected among all plant samples was used as the background value (items·g^−1^). C_i_ represents the MP abundance at a given sampling site; PLI_n_ denotes the pollution load index of MPs at a given sampling site; and n is the sampling site number. PLI represents the overall MP pollution load index of the study area. The classification criteria for both indices are provided in the Supplementary Materials, Table S2.

### 4.8. Data Analysis

Statistical analyses were performed using Microsoft Excel 2021 and SPSS 29.0. Spatial distribution maps of the sampling sites were generated using Adobe Photoshop 2024. Other figures were produced using Origin 2021, SmartPLS 4, and R 4.4.1.

Based on MP abundance data in forest soils, the values of the BCF and TFs for different bamboo components were calculated. Pearson correlation coefficients were computed using standardized variables, and two–tailed significance tests were applied to assess statistical significance. Correlation heatmaps were generated to evaluate the relationships among root morphological traits, soil chemical properties (Supplementary Materials, Table S3), soil physical properties (Supplementary Materials, Figure S2), and soil MP characteristics (Supplementary Materials, Figure S1), as well as their effects on MP bioaccumulation and translocation factors in rhizome–root systems and bamboo shoot components.

## 5. Conclusions

This study demonstrated that urban–suburban distance regulates MP accumulation and distribution in the rhizome–root systems and bamboo shoots of mulched *Ph. violascens* forests. MP abundance was higher in suburban forests, and the distribution patterns differed between forest types: MPs were mainly enriched in rhizome roots and bamboo rhizomes in suburban forests, whereas enrichment in exurban forests was primarily observed in rhizome roots and bamboo shoots. Long–term mulching further intensified MP accumulation, particularly in belowground components. MPs were dominated by small, particulate forms, while larger particles were mainly retained in root structures, indicating size–selective transport. Dominant polymers (PE, PET, PP, ACR, and PVC) were consistently detected, suggesting stable sources and transport pathways. MP translocation exhibited stage–specific and non–linear characteristics, with urban–suburban gradients mainly influencing early uptake and transfer processes. Soil pH and total potassium, together with plant structural differentiation, jointly regulated MP migration. However, these findings are primarily based on correlational analyses, and the ecological risk indices represent indirect proxies derived from polymer composition and abundance, lacking direct toxicological evidence and mechanistic validation of MP transport processes.

## Figures and Tables

**Table 1:** Morphological characteristics in bamboo rhizome roots (BRRs) and bamboo clump roots (BCRs) of *Ph. violascens*.

Experimental Forest Types	Bamboo Root Components	Total Root Length RL (cm)	Root Surface Area RSA (cm^2^)	Root Diameter RD (mm)	Specific Root Length SRL (cm·g^−1^)	Root Volume RV (cm^3^)	Root Tip Number RTN	Root Branching Number RBN	Root Crossing Number RCN
SB0	BCR	36.82 ± 16.63 Bb	28.77 ± 2.41 Aa	0.26 ± 0.09 Aa	7.36 ± 3.33 Bb	2.08 ± 1.24 Aa	267.43 ± 35.85 Ba	936.60 ± 193.53 Aa	172.57 ± 4.69 Aa
	BRR	66.50 ± 34.00 Ba	20.31 ± 12.61 Ba	0.13 ± 0.04 Ba	13.30 ± 6.80 Ba	0.50 ± 0.36 Ca	360.80 ± 197.77 Ba	1064.24 ± 626.98 Ba	146.03 ± 90.00 Ca
SB2	BCR	36.86 ± 19.19 Bb	22.33 ± 2.79 Ba	0.20 ± 0.11 Ba	7.37 ± 3.84 Bb	1.34 ± 0.96 Ba	247.99 ± 47.84 Cb	751.76 ± 161.94 Cb	118.42 ± 15.09 Ba
	BRR	47.70 ± 23.88 Cb	29.67 ± 22.16 Aa	0.26 ± 0.19 Aa	9.54 ± 4.78 Cb	1.92 ± 2.00 Aa	366.22 ± 193.63 Ba	1024.77 ± 541.43 Ba	220.28 ± 153.15 Ba
SB4	BCR	73.02 ± 22.78 Ab	17.20 ± 5.87 Cb	0.13 ± 0.01 Cb	14.60 ± 4.56 Ab	0.34 ± 0.16 Cb	324.43 ± 114.65 Ab	889.99 ± 303.57 Bb	104.72 ± 45.35 Cb
	BRR	83.86 ± 41.47 Aa	29.73 ± 26.20 Aa	0.22 ± 0.19 Aa	16.77 ± 8.29 Aa	0.96 ± 1.32 Ba	480.38 ± 266.87 Aa	1352.25 ± 957.94 Aa	251.06 ± 215.88 Aa
EB0	BCR	43.47 ± 8.24 Ca	10.39 ± 4.17 Cb	0.07 ± 0.02 Bb	8.70 ± 1.65 Ca	0.20 ± 0.12 Cb	200.68 ± 15.45 Cb	599.81 ± 182.99 Cb	75.13 ± 16.09 Cb
	BRR	30.25 ± 13.77 Cb	6.96 ± 2.85 Cb	0.05 ± 0.02 Cb	6.05 ± 2.76 Cb	0.13 ± 0.05 Cb	142.82 ± 67.77 Cb	362.70 ± 186.48 Cb	51.76 ± 31.35 Cb
EB2	BCR	53.45 ± 32.22 Ba	13.93 ± 9.37 Bb	0.08 ± 0.04 Bb	10.69 ± 6.44 Bb	0.31 ± 0.22 Bb	300.88 ± 169.16 Ba	801.25 ± 565.37 Ba	119.37 ± 74.86 Ba
	BRR	73.83 ± 42.52 Aa	17.08 ± 9.43 Ab	0.13 ± 0.08 Ab	14.77 ± 8.50 Ab	0.32 ± 0.17 Ab	348.80 ± 228.12 Ab	918.56 ± 546.35 Ab	123.81 ± 70.87 Ab
EB4	BCR	131.54 ± 93.18 Aa	36.06 ± 31.80 Aa	0.21 ± 0.12 Aa	26.31 ± 18.64 Aa	0.85 ± 0.96 Aa	600.55 ± 455.47 Aa	1592.61 ± 1076.06 Aa	248.20 ± 201.32 Aa
	BRR	44.79 ± 27.63 Bb	10.94 ± 6.15 Bb	0.09 ± 0.05 Bb	8.96 ± 5.53 Bb	0.22 ± 0.11 Bb	205.40 ± 141.18 Bb	551.37 ± 370.25 Bb	71.32 ± 48.53 Bb

Note: (1) Different uppercase letters indicate significant differences for a given parameter under the same urban–suburban distance (i.e., across different mulching durations and bamboo components) (*p* < 0.05); (2) different lowercase letters indicate significant differences for a given parameter under the same mulching duration (i.e., across different urban–suburban distances and bamboo components) (*p* < 0.05).

**Table 2:** Ecological risk index (H) and pollution load index (PLI) of MPs in bamboo shoots from suburban and exurban mulched *Ph. violascens* forests.

Experimental Bamboo Forests	Ecological Risk Index (H)		Pollution Load Index (PLI)	
SB0	2861.86	IV	1.22	Moderate pollution
SB2	639.63	III	1.02	Moderate pollution
SB4	5718.68	IV	1.41	Moderate pollution
EB0	4453.11	IV	1.62	Moderate pollution
EB2	1561.27	IV	1.46	Moderate pollution
EB4	6876.81	IV	1.00	Moderate pollution

## Data Availability

The raw data supporting the conclusions of this article will be made available by the authors upon request.

## Supplementary Materials

The following supporting information can be downloaded at: https://www.mdpi.com/article/10.3390/plants15111690/s1, Table S1: Hazard scores of different polymer types; Table S2: Classification of ecological risk levels for MPs; Table S3:Variations in soil physicochemical properties in experimental Ph. violascens forests; Figure S1: Abundance and vertical distribution of soil microplastics (MPs) in experimental *Phyllostachys violascens* forests; Figure S2: Proportions of soil aggregate size fractions at different soil depths in experimental *Ph. violascens* forests.
