# Supplementary material for: Spatial Distribution and Compartmental Allocation of Microplastics in Belowground Systems of Mulched Phyllostachys violascens Forests Along Urban–Rural Gradients
Source: Plants (Basel). 2026 May 30;15(11):1690. doi: 10.3390/plants15111690 (PMC13259302; doi:10.3390/plants15111690)
Supplement: Supplementary file 1 [file plants-15-01690-s001.zip › Supplementary Materials-Figure.pdf]

List of Supplymental Figures

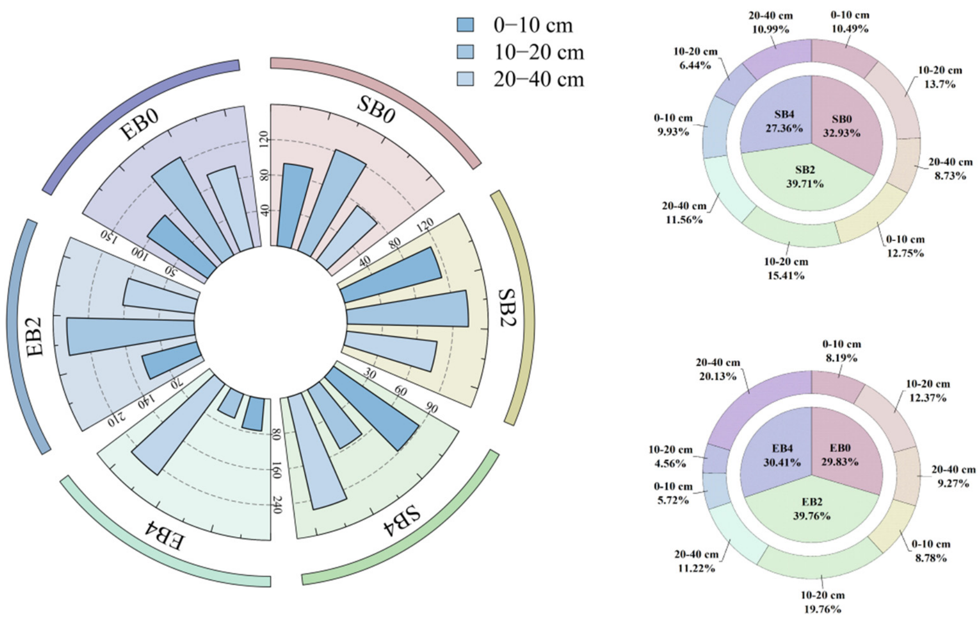

Figure S1 Abundance and vertical distribution of soil microplastics (MPs) in experimental Phyllostachys violascens forests

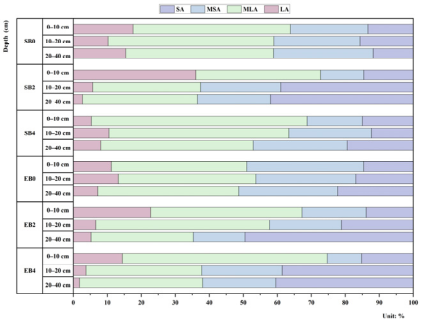

Figure S2 Proportions of soil aggregate size fractions at different soil depths in experimental Ph. violascens forests

Note: SA, small aggregates; MSA, medium –small aggregates; MLA, medium –large aggregates; LA, large aggregates. SB0, Suburban site, no mulch (0 years); SB2, Suburban site, short – term mulch (2 – years); SB4,Suburban site, long – term mulch (4 – 6 years); EB0, Exurban site, no mulch (0 years); EB2, Exurban site, short – term mulch (2 – 3 years); EB4, Exurban site, long – term mulch (4 – 6 years).
